# Risk perceptions of avian influenza among poultry farmers on smallholder farms along border areas of Thailand

**DOI:** 10.3389/fvets.2023.1075308

**Published:** 2023-02-08

**Authors:** Soawapak Hinjoy, Pornchai Thumrin, Jitphanu Sridet, Chat Chaiyaso, Punnarai Smithsuwan, Janjao Rodchangphuen, Yupawat Thukngamdee, Weerachai Suddee

**Affiliations:** ^1^Department of Disease Control, Ministry of Public Health, Nonthaburi, Thailand; ^2^Department of Livestock Development, Bangkok, Thailand

**Keywords:** avian influenza, risk perception, community, Thailand, poultry farmers on small farms

## Abstract

**Introduction:**

Thailand has not reported any cases of avian influenza since 2008. However, avian influenza viruses circulating in poultry in neighboring countries may have potential for transmission to humans. The aim of this study was to assess risk perceptions of poultry farmers and traders in three border provinces of Thailand adjacent to Laos.

**Materials and methods:**

Poultry farmers and traders were interviewed in-person during October–December 2021 by health and livestock officials using a standardized questionnaire to collect demographics, job histories, knowledge, and practices related to avian influenza. Knowledge and practices were scored using 22 questions with a 5-point scale. Exploratory data analysis scores above and below the 25th percentile was used as the cut-off point for perception scores. The cut-off point was used to describe perceptions of respondent characteristics in order to compare differences between groups with more or < 10 years of experience. Age adjusted perceptions of disease risk were analyzed by multivariable logistic regression.

**Results:**

Of the 346 respondents, the median risk perception score was 77.3% (22 questions with a 5-point scale, so the total score was 110). Having more than 10 years of experience in poultry farming was significantly associated with an increased perception of the risk of avian influenza (adjusted odds ratio 3.9, 95% confidence interval 1.1–15.1). Thirty-two percent of participants perceived avian influenza as a risk only during the winter season, and more than one-third of the participants (34.4%) had not received recent information about new viral strains of avian influenza.

**Discussion:**

Participants did not perceive some key information on the risks associated with avian influenza. Regular training on the risks of avian influenza could be provided by national, provincial and/or local officials and they, in turn, could share what they learn with their communities. Participants who had greater experience in poultry farming were associated with greater risk perception. Experienced poultry farmers and traders working on poultry farms can be a part of the community mentorship program to share their experiences and knowledge on avian influenza with new poultry producers to improve their perception of disease risk.

## Introduction

Avian influenza is an infectious disease caused by influenza type A viruses in the Orthomyxoviridae family, which cause infections in both humans and many kinds of animals such as horses, pigs, cats, birds, and chickens. The disease in animals, especially in poultry, has been detected for >100 years, with occasional outbreaks in countries such as England, Canada, Australia, the United States, Mexico and Italy (1). Avian influenza viruses are generally not highly contagious to humans (2). The first evidence of animal-to-human transmission was reported when a highly pathogenic avian influenza (HPAI) A(H5N1) virus was transmitted to humans in the Hong Kong Special Administrative Region in 1997 (3). Humans are mainly infected with avian influenza A(H5N1) through poultry according to available epidemiological data (4, 5). In late 2003–2004, avian influenza detected in Thailand and neighboring countries including Cambodia, Laos, and Malaysia (1). The Division of Epidemiology in the Thai Ministry of Public Health received reports of and investigated 25 human cases of influenza A(H5N1) virus infection, including 17 deaths, from 2004 to 2006. In 2006, the year that the last avian influenza A(H5N1) virus outbreak among humans in Thailand was reported, there were three persons with confirmed infection, and all of three died (2). With respect to the high case fatality rate of avian influenza A(H5N1) virus, this zoonosis continues to be a priority for disease prevention in Thailand.

During the influenza A(H5N1) outbreaks in Thailand during 2004–2006, the Thai Department of Livestock Development implemented measures that included culling flocks that had infected birds by the veterinary authorities. Nationwide surveillance program of HPAI infections and active surveillance for avian influenza virus in poultry to control avian influenza outbreaks and to monitor the situation of avian influenza in Thailand has been implemented continuously since 2006 (4, 6). Although Thailand has not reported avian influenza A (H5N1) in poultry for more than 16 years, there is continued risk of avian influenza outbreaks. The World Organization for Animal Health (WOAH, formerly called OIE) has reported that cases of severe and mild avian influenza infections have occurred in avian populations and in people living in countries in the same region as Thailand (7).

Live poultry traders and poultry farmers may be at increased risk for avian influenza infections due to factors including the duration of time working in close contact with poultry and behaviors that may pose a risk of exposure to pathogens (8). A study by Dikky et al. (9) supported using data obtained from surveys on the behavior of personnel in the poultry industry to inform disease control measures. Identifying which people influence attitudes, knowledge and beliefs regarding avian influenza control in the community can help reduce the spread of avian influenza in the area (10). The aim of this study was to assess the risk perceptions of traders and farmers in the poultry trade network along border provinces of Thailand. Knowledge on the perceived risk of avian influenza infection in live poultry traders and poultry farmers in the study areas can inform risk communication guidelines and facilitate effective avian influenza prevention practices in the context of the country and the region.

## Methods

Target provinces are located along borders with countries that have reported avian influenza outbreaks in recent years. The study was conducted in all sub-districts of three districts in Nakhon Phanom, Mukdahan and Ubon Ratchathani provinces (one district was selected in each of the three provinces). There was a registered population of chickens and ducks with a number of poultry farmers in three provinces under the Department of Livestock Development; 3,065,744 chickens and ducks, and 7,780 farmers in Ubon Ratchathani, 320,141 chickens and ducks, and 4,403 farmers in Nakhon Phanom, 143,661 chickens and ducks, and 1,097 farmers in Mukdahan. The majority of participants were small-scale poultry operations. Mixed-type poultry means raising several types of poultry on the same farm, such as broiler chickens, layer chickens, fighting cocks and ducks on the same farm. These three provinces were chosen because of the on-going active surveillance of poultry farms and trades in the areas. In addition, international movements of poultry were reported in these areas. Persons targeted to participate were poultry farmers and poultry traders who raised and contacted (for example, holding, feeding, culling) at least one bird on their farms or backyards. The inclusion criteria were poultry traders and farmers aged 18 years and older with the ability to listen, speak and read the Thai language. The study population was required to have lived in the study area for at least 1 year prior to participating in the study. The exclusion criteria for participation were not having been involved in poultry operations for >1 month before being enrolled in the study and not being included in the Provincial Livestock Office's registration database in 2019. Due to the limited number of poultry traders in the study areas, all poultry traders listed in the Livestock District Office database in each district were eligible to participate. The sample size of poultry farmers was calculated using the formula from the Tool 5 value chain sampling guidelines (11). The previous study in Karachi, Pakistan revealed that the prevalence of avian influenza viruses in commercial layers was 26.45% and prevalence of H9 virus was 40.16% (12). With respect to avian influenza, including high pathology and low pathology, however, we had no accurate data on low pathological avian influenza viruses in Thailand. The risk of avian influenza has therefore been estimated at 50% of the total population. Precision was set at 7.5% with a z-score of 1.645. The sample size was calculated to be 112 poultry farmers per district using random sampling in each sub-district. A simple random sampling of the poultry farm addresses and the poultry trader addresses from the livestock district office database was conducted based on the 2019 District Livestock Office database of poultry farmers in the three provinces using Epi Info (13). All eligible participants selected from the random sampling were invited to participate in the study through a letter informing them of the requirements. All participants were asked to sign a written informed consent document. All study procedures were reviewed and approved by the Ethics Committee for Research in Human Subjects, Department of Disease Control, Ministry of Public Health, Thailand (number FWA 00013622) on November 2, 2021.

After written informed consent was obtained, all participants answered a standardized questionnaire. The questions consisted of demographics, knowledge, attitude and practices on avian influenza for example, knowledge about severity of symptoms of avian influenza, zoonotic strain of avian influenza, route of transmission, importance of spraying disinfectant on vehicles going across the farms, practices while moving in and out the poultry in the areas, practices of separation of diseased poultry from healthy poultry in the herd, practices of raising poultry on the farms, destroying the unknown cause of death of poultry, wearing a mask while working on a poultry farm, and notification to the relevant authorities about unusually sick or dead birds. All information were recorded in the face-to-face interview. The interview process was conducted by trained health and veterinarian officers.

Exploratory data analysis and statistical analysis were performed using Epi Info (13). Two-sided *p* < 0.05 were considered to be statistically significant. Knowledge and practices were scored using 22 questions with a 5-point scale, so the maximum possible score was 110. The exploratory analysis of the data assessed the scores above and below the 25th percentile as the cut-off point for the risk perception scores. A reason for the 25th percentile was the data distribution during exploratory data analysis that the distribution was much left skewed. Simple tabulation was used to describe proportions of risk perception scores (cut-off scores at ≥25th percentile or < 25th percentile) in each category of exposure variables for example, respondents were divided into two groups based on years of experience in poultry farming/trading (< 1 year vs. >1 year's experience).

Univariate analysis was performed by calculating odds ratios (ORs) and 95% confidence intervals (CIs) to evaluate each risk factor for the risk perceptions. In order to account for confounding factors, multiple logistic regression analysis was performed. Backward elimination procedure was used in the model. Changes of 10% in coefficients were considered evidence of possible confounding. Any variables that remained significant were kept in the model. Adjusted ORs and 95% CIs were also calculated.

## Results

There were 346 participants, all of whom were classified as domestic breeders or poultry farmers on small-scale poultry farms primarily for their own domestic use in local areas, including 338 farmers (97.7%) and eight persons (2.3%) who worked in both poultry farming and poultry trading jobs. The proportions of females and males were almost equal (184 females [53.2%] and 162 males [46.8%]). The ages of participants ranged from 18 to 78 years old. The mean and median ages were 50 years. Most of the participants (96.8%) did not have a bachelor's degree. The participants' monthly incomes were mostly under 10,000 Baht (i.e., under 300 USD). In Table 1, most poultry farmers were classified on small farms that had less than 10% of poultry farmers with over 100 poultry on their farms. Among the poultry farmers, 120 (34.7%) raised mixed-type poultry. In mixed-type poultry, there were mixed backyard poultry on the farm in a number of 103 farms, 54 farms had duck or geese mixed on the farm and 45 farms had fighting cocks on the farm. There were 171 farms that raised only backyard poultry, 47 farms with only fighting cocks, and 8 farms with only ducks.

**Table 1:** Husbandry practices of poultry farmers along border areas in three provinces of Thailand.

**Characteristics/husbandry practices**	**Nakhon Phanom**	**Mukdahan**	**Ubon Ratchathani**
**Total number of poultry on the farm**			
2–40	54 (48.2)	71 (60.2)	59 (51.3)
41–100	46 (41.0)	37 (31.4)	46 (40.0)
101–530	12 (10.7)	10 (8.5)	10 (8.7)
**Farmers agree to obtain permission to move the poultry with the livestock agent before moving the poultry out of the area**			
No	28 (25.0)	14 (11.9)	72 (20.9)
Yes	84 (75.0)	104 (88.1)	273 (79.1)
**Farmers understand the importance of quarantine new poultry before they are raised with the herd**			
No	13 (11.7)	10 (8.5)	12 (10.4)
Yes	98 (88.3)	108 (91.5)	103 (89.6)
**Farmers understand that it is important to notify the relevant authorities if they detect abnormally sick or dead birds**			
No	58 (51.8)	63 (53.4)	34 (29.6)
Yes	54 (48.2)	55 (46.7)	81 (70.4)
**Farmers understand that it is important not to destroy the unknown cause of death of poultry by pouring them into rivers**			
No	8 (7.1)	23 (19.5)	11 (9.6)
Yes	104 (92.9)	95 (80.5)	104 (90.4)
**Farmers agree to wear a mask while working on a poultry farm to reduce the risk of avian influenza infection**			
No	15 (13.4)	12 (10.2)	8 (7.0)
Yes	97 (86.6)	106 (89.8)	107 (93.0)
**Farmers understand that they are not selling abnormally sick and dead poultry as usual**			
No	3 (2.7)	6 (5.1)	3 (2.6)
Yes	109 (97.3)	112 (94.9)	112 (97.4)

Most farmers (>75%) in three provinces have a good understanding of good practices aimed at reducing the risk of avian influenza, for example; obtaining permission to move the poultry with the livestock agent before moving the poultry out of the area, the importance of quarantine new poultry before they are raised with the herd, the importance of not destroying the unknown cause of death of poultry by dumping them into rivers, the importance of wearing a mask while working on a poultry farm and understand not to sell abnormally sick and dead poultry as shown in Table 1. However, less than 50% of farmers in Nakhon Phanom and Mukdahan provinces did not see the importance of informing the relevant authorities if they detected abnormally sick or dead poultry (Table 1).

A total of 183 (52.9%) participants stated they had over 10 years of experience in poultry farming or trading. There were only 10 participants who had been in the poultry industry for < 1 year. Most participants (72.5%) had < 1 h of contact with poultry per day.

The scores for correct responses about perceived risk on avian influenza among live-poultry traders and farmers on the questionnaire ranged from 51.1 to 96.6%. The average and median risk perception scores were 76.7 and 77.3%, respectively. Participant characteristics were classified into two levels of risk perception scores, those above and those below the 25th percentile, as shown in Table 2.

**Table 2:** Risk perception scores on avian influenza by participant characteristics among live-poultry traders and farmers along border areas in three provinces of Thailand.

**Characteristics**	**No. (%) under 25th percentile of risk perception scores**	**No. (%) above 25th percentile of risk perception scores**	***p*-value**
**Location (province)**			0.80
Nakhon Phanom	34 (33.0)	69 (66.9)	
Mukdahan	35 (31.3)	77 (68.8)	
Ubon Ratchathani	38 (35.2)	70 (64.8)	
**Sex**			0.19
Female	63 (36.2)	111 (63.8)	
Male	44 (29.3)	106 (70.7)	
**Occupation**			0.79
Poultry farmer	104 (32.9)	212 (67.1)	
Both poultry farming and poultry trading jobs	3 (37.5)	5 (62.5)	
**Age (years)**			0.04
18–43	28 (30.1)	65 (69.9)	
44–58	46 (29.1)	112 (70.9)	
59–78	33 (45.2)	40 (54.8)	
**Educational level**			
Below bachelor's degree	104 (33.2)	209 (66.8)	0.68
Bachelor's degree or higher	3 (27.3)	8 (72.7)	
**Monthly income (Baht)**			0.08
< 10,000	91 (14.3)	163 (85.7)	
10,001–20,000	14 (25.0)	42 (75.0)	
>20,000	2 (14.3)	12 (85.7)	
**Years of experience in poultry farming/trading**			0.16
< 1	6 (60.0)	4 (40.0)	
1–10	45 (31.5)	98 (68.5)	
>10	56 (32.8)	115 (67.3)	

There were no significant differences in risk perception scores in different study areas or between poultry farmers and traders. The number of women with risk perception scores below the 25th percentile was higher than men and those who did not hold a bachelor's degree. However, after adjusting for potential factors, no differences were found in the risk perception scores for the variables of sex and educational level. The number of study participants who had risk perception scores below the 25th percentile was observed to be higher among older adults, but this association was not significant. There was no difference in risk perception scores based on monthly income. Having >10 years of experience in poultry farming/trading was independently associated with increased risk perception scores (OR = 3.89, 95% CI = 1.09–15.07) (Table 3).

**Table 3:** Multivariate logistic regression analysis of factors associated with risk perception scores on avian influenza among live-poultry traders and farmers along border areas in three provinces of Thailand.

**Variable**	**Crude odds ratio (95%CI)**	***p*-value**	**Adjusted odds ratio (95%CI)**	***p*-value**
**Age group (years)**				
18–43	Reference			
44–58	1.05 (0.60–1.84)	0.87	1.05 (0.58–1.90)	0.88
≥59	0.53 (0.28–0.99)	0.05	0.51 (0.25–1.06)	0.07
**Experience in poultry farming/trading**				
< 1 year	Reference			
1–10 years of experience in poultry farming/trading	3.08 (0.84–11.4)	0.09	3.70 (0.95–14.43)	0.05
>10 years of experience in poultry farming/trading	3.27 (0.88–12.15)	0.08	3.89 (1.09–15.07)	0.04

The measures for which the most frequent respondents (over 95%) responded correctly to prevent and control avian influenza were as follows: understanding the need to avoid the consumption, sale and sudden feeding of dead poultry on farms to other animals, due to the risk of avian influenza. In addition, the measures for which the participants most commonly provided incorrect responses to prevent and control avian influenza were: unclear threat from of avian influenza, avian influenza high risk only in wintertime, and lack of knowledge of new viral strains.

## Discussion

In this study population, the median risk perception score was high. This result may be because of heightened knowledge and awareness following the 2004–2006 avian influenza A(H5N1) outbreak in Thailand, in which there were in 25 human cases recorded with 17 deaths (9). After the outbreaks during 2004–2006, many organizations launched public awareness campaigns about the impact of avian influenza. Therefore, people, farmers and traders in Thailand may have had increased access to information and become more aware of the risk of avian influenza.

Risk perception scores indicate that the study population had reasonably good awareness of avian influenza. Their awareness may have resulted from the experience of the previous avian influenza outbreak (10) and information from the avian influenza surveillance network along the border between Thailand and Laos (14). The information obtained through surveillance has enabled poultry farmers to receive the current avian influenza situation that they may be aware of to prevent avian influenza and keep their poultry safe. In 2005, the results of a European and Asian avian influenza found only moderate perceptions of risk compared to this study (10). A total of 3,436 respondents were interviewed participation in the study of a European and Asian avian influenza risk perception. The perception varied from 32% in Denmark and Singapore to 61% in Poland and Spain. Higher scores were observed in Europe than in Asia.

Huge impact of economic loss during avian flu epidemics in Thailand from the mass culling of over 1-billion-baht poultry as compensation to affected owners (15). According to data from the United States Department of Agriculture (USDA) in April 2022, Thailand is the world's sixth largest chicken producer and the world's third largest chicken exporter (16). This may be one reason why the public-private partnership continues to promote a higher perception of the risk of avian influenza in Thailand. The public and private sectors need to continually support collection of information and sharing of knowledge to enhance public relations on avian influenza prevention. This would help ensure that poultry farmers have better understanding of the disease and a higher level of perceived risk. The continued engagement of government and private organizations is a key factor in maintaining awareness of avian influenza in communities (6). Knowledge, attitudes and best practices among poultry farmers and traders are critical to preventing the spread of avian influenza in humans and animals.

This study found that sex and age were not associated with perceived risk and avian influenza prevention and control. That is consistent with the study by Vityakom and Chayyaphong (17). In Table 3, few women were aware of risk, compared to men, but this association was not significant. A study by Cui et al. (18) concluded that a high perception and awareness of the risk of a disease were positively correlated with willingness to practice protective behaviors to prevent avian influenza A(H7N9) infection. The community education program may be more targeted to women because the perception of risk among women may influence other family members in the household.

In Table 3, most subjects over 60 years old had low aggregate scores on perceived risks of avian influenza compared with other age groups. Our study revealed that, based on univariate analysis, older adults had a lower perception of avian influenza risk, similar to a study by Fielding et al. (19). Suggesting that they underestimated the hazards and consequences due to familiarity with the hazards and past experience, they viewed the current avian influenza outbreak as a low risk. The study by Chesser et al. (20) reported that older adults may have additional issues with memory and perception, which could reduce their perception of health risks. In addition, the study by Louie et al. (21) found that patients aged 50–59 years had a higher mortality rate due to respiratory diseases such as influenza A(H1N1). Appropriate self-care behaviors to prevent infection can decrease the severity and complications of respiratory diseases, like avian influenza which can have serious consequences, especially for the elderly (22).

The elderly in the study population had less awareness of the risk of avian influenza. It is possible that this age group may neglect to take care of their own health and may take care of sick poultry in the farm or around the house without taking precautions. If they contract avian influenza without knowing, they may delay seeking medical advice. Increasing the risk perceptions among older groups is important to avoid underestimating avian influenza in this group. In terms of basic hygiene, this study found that most elderly people have good knowledge and practices in hand-washing. Perhaps our study was conducted during the COVID-19 pandemic and most people became more aware of the importance of masks and hand washing practices.

The scores from poultry farmers and traders in our study showed that more experience was significantly associated with an increased risk perception of avian influenza [adjusted odds ratio (95%CI) 3.89 (1.09–15.07)] as shown in Table 3. These results suggest that the participants in this study who had more years of experience in poultry farming had a more realistic understanding of the risks of avian influenza. This is similar to a study by Asare et al. which showed that work experience affects the perception and knowledge of avian influenza in poultry workers in Ghana (23). In addition, it was in accordance with the study by Cui et al. that showed an association between risk perceptions and personal protective behaviors on poultry farms in China. The study found that the number of years of poultry farming were significantly associated to personal protection behaviors and biosecurity prevention behaviors (24). Many factors can influence the perception of disease risk, including individuals' backgrounds, past experiences, availability of the source of information, social context and individual interpretation. Education and learning new information play a significant role in improved individual health knowledge as shown in the study by Pawun et al. (25). An approach to help enhance awareness and understanding of the risks of avian influenza of new poultry producers is to have a community platform for the more experienced poultry farmers to share experiences and specific knowledge. Government officials or local livestock officials may consider implementing various forums for sharing experiences and knowledge as part of the community mentorship program.

According to the World Health Organization's Avian Influenza Situation Report, people infected with the avian influenza virus tend to have a history of contact with poultry or have visited live poultry markets (26). Selling poultry sick/dead of unknown reasons, especially in live poultry markets, is a significant risk factor for the spread of avian influenza in humans and in poultry flocks (27). This knowledge is particularly important to help reduce the spread of the avian influenza virus. It is good to know that poultry farmers and traders in this study had high risk perception scores to prevent and control avian influenza. Most of the participants understand that sick/dead poultry should not be sold and consumed and that they should not be used for animal feed. Knowledge such as the severity of the disease, the pathogenic strains of avian influenza and the seasonal variation of the disease may not be understood among poultry farmers and traders, as shown in the results. This may be because Thailand has not reported an outbreak of avian influenza for >16 years, resulting in lack of knowledge of information on avian diseases. Increasing risk communication to officials, the poultry industry and the public about avian influenza is a necessary strategy in line with strengthening surveillance, prevention and control of avian influenza.

This study has some limitation of bias, including a very small number of traders and fewer people with less than a year of farming experience to recruit into the study. This study used the Provincial Livestock Office's registration database as a sampling frame. Poultry farmers and traders who was not being in the Provincial Livestock Office's registration database in 2019 had been excluded from this study. Of the non-registered farmers and traders not included in this study, they may have different characteristics with the registered farmers and traders. Therefore, bias may be present in this study. All farmers came from a small poultry network, some of which held positions as farmers and traders. The poultry trader could not be split into one particular category. In addition, our study focused on small poultry farms so that results could not refer to large industrial farms.

In conclusion, the transfer of knowledge and practices from experienced poultry farmers to individuals newer in the poultry industry at the community level is a good strategy. The community mentorship program to share experiences and knowledge on avian influenza can increase the risk of disease perception through effective communication among farmers. An accurate information and awareness of avian influenza of poultry farmers can reduce the risk of contracting and spreading the avian influenza virus in the community.

## Data availability statement

The raw data supporting the conclusions of this article will be made available by the authors, without undue reservation.

## Ethics statement

The studies involving human participants were reviewed and approved by Ethics Committee for Research in Human Subjects, Department of Disease Control, Ministry of Public Health, Thailand (number FWA 00013622) on November 2, 2021. The patients/participants provided their written informed consent to participate in this study.

## Author contributions

Conceptualization and methodology: SH and WS. Investigation and data collection: PT, JS, CC, PS, JR, YT, and WS. Writing—original draft preparation: SH. Reviewing and editing: SH, PT, and WS. All authors have read and agreed to the published version of the manuscript.
